# Farm environments: Reservoir for Multidrug‐Resistant and Virulent *Escherichia coli* in Bangladesh

**DOI:** 10.1155/ijm/3646267

**Published:** 2026-10-29

**Authors:** Elias Mia, Tasnimul Arabi Anik, Afroja Yasmin, Humaira Akhter, Anowara Begum

**Affiliations:** ^1^ Environmental Microbiology Laboratory Department of Microbiology University of Dhaka Dhaka Bangladesh du.ac.bd; ^2^ Department of Pathology Faculty of Veterinary Medicine and Animal Science Gazipur Agricultural University Gazipur Bangladesh

**Keywords:** antimicrobial resistance, *Escherichia coli*, farm animal, multidrug-resistant phenotype, pathotypes, phylogrouping

## Abstract

Farm environments, especially dairy and poultry farms, are the major sources of multidrug‐resistant (MDR) pathogenic *Escherichia coli*. At present, knowledge regarding the prevalence of MDR *E. coli*, phylogroup, and pathotype in various farm environments of Bangladesh is inadequate. Highlighting the issue, the present investigation was carried out to determine the prevalence of MDR *E. coli*, phylogroup distribution, and pathotype diversity in various farm environments. Thirteen types of samples were collected from poultry, dairy, and aquaculture in Cumilla, Bangladesh, during three seasons (rainy, summer, and late autumn). Following identification by cultural, biochemical, and molecular techniques, the isolates were investigated for AMR profile, ESBL production, biofilm‐forming capability, antibiotic resistance, virulence and adhesin genes, and phylogroup pattern. Sixty‐three *E. coli* isolates, mostly from dairy and poultry, were identified from the collected samples. Among the 19 antibiotics tested, resistance to ciprofloxacin (55.54%) was the most common, whereas all the isolates were sensitive to ceftazidime (30 *μ*g). The MAR index and the percentage of MDR isolates were higher in the rainy season samples than in the summer. Between sources, the prevalence of MDR isolates was more common in poultry (78.79%) than in cattle farms (25%). Poultry isolates showed a significantly higher frequency of antibiotic resistance genes than dairy isolates (*p* − value < 0.05). In the biofilm‐forming phenotypic test, 93.7% of the isolates were biofilm formers. The biofilm‐forming ability was higher in Phylogroup B1 than in Phylogroup C (*p* − value < 0.05). Among the phylogroups, B1 (73.02%) was the most common, followed by C (19.02%) and A (3.17%). Almost 50.7% of isolates were pathogenic with EHEC (34.92%) being most prevalent. The most common resistance genes were *tetA*, *qnrS*, *sul-2*, *dfrA17*, *dfrA1*, *blaSHV*, and *blaTEM*. These results emphasize the need for focused surveillance and mitigation efforts to control the spread of MDR *E. coli* in farm environments, especially in poultry.

## 1. Introduction

Antibiotic‐resistant bacteria (ARB) in the environment are a growing global public health hazard in the 21st century. Livestock and poultry farms have been the major sources of ARB because of the improper and excessive use of antibiotics to keep the livestock disease‐free [1, 2]. One significant pathogen that often acts as a vector for antimicrobial resistance is *Escherichia coli* [3]. Studies have revealed that *E. coli* strains associated with animals often demonstrate resistance to various antibiotics such as carbapenem, minocycline, doxycycline, gentamicin, sulfamethoxazole–trimethoprim, erythromycin, chloramphenicol, *β*‐lactams, fluoroquinolones, third‐generation and fourth‐generation cephalosporins [3–6]. A recent study reported a high prevalence of resistance in *E. coli* strains isolated from a poultry farm, with nearly 64.6% of isolates demonstrating resistance to multiple antibiotic classes [7]. A similar scenario exists in dairy, where approximately 74.4% of *E. coli* isolates from small‐scale dairy cattle in Tanzania showed multidrug resistance to three to eight classes of antimicrobials, with considerable resistance to ampicillin and cefotaxime [8]. In a study conducted in 2023, all *E. coli* strains isolated from milk samples were multidrug‐resistant (MDR) [9]. Resistance in *E. coli* is often mediated by the production of beta‐lactamases, particularly extended‐spectrum beta‐lactamases (ESBLs), which promote resistance to penicillins and cephalosporins [10]. A recent study reported a prevalence of 88.8% for ESBL‐producing *E. coli* [11]. These enzymes are typically encoded on plasmids, facilitating horizontal gene transfer between bacteria. This is a considerable worry, particularly in underdeveloped and developing countries, where the misuse and overuse of antibiotics are common due to a lack of strict regulations. Continuous exposure to antibiotics creates selective pressure that promotes the survival of resistant strains [12]. These resistant bacteria can then easily spread to the environment, especially since untreated farm waste is often disposed of in open places, such as agricultural sites [13].

Although most *E. coli* strains are harmless commensals, the spread of resistant *E. coli* isolates from domestic animals can pose a significant threat, particularly to immunocompromised individuals. Even in healthy individuals, the bacteria can lead to serious infections, including enteritis, septicemia, urinary tract infection, and diarrhea. The degree of severity of an infection depends on several virulent factors present in different strains of pathogenic *E. coli*. Pathogenic *E. coli* are usually classified into two categories: diarrheagenic *E. coli* (DEC) and extraintestinal pathogenic *E. coli* (ExPEC) [14]. Diarrheal illnesses are often caused by DEC, one of the most common bacterial agents of diarrhea [15]. Infections caused by DEC are a major public health concern, accounting for a significant proportion of acute diarrhea episodes in children under 5 years of age in developing countries [15].

The DEC consists of several pathotypes that are characterized by the presence or absence of specific virulence genes. In enterotoxigenic *Escherichia coli* (ETEC), the *eltB* gene encodes heat‐labile enterotoxin (LT), which increases fluid secretion in the intestines, whereas *estA* encodes heat‐stable enterotoxin (ST), promoting diarrhea. In enteropathogenic *Escherichia coli* (EPEC), the *eaeA* gene encodes intimin, an adhesin that facilitates attachment to intestinal epithelial cells, and *bfpA* encodes bundle‐forming pili that aid in adherence. In EHEC, the *eaeA* gene is involved in adherence, whereas *vt1* and *vt2* encode Shiga toxins that damage the intestinal lining. In enteroinvasive *Escherichia coli* (EIEC), the *ial* gene encodes an invasion‐associated locus that allows the bacteria to invade intestinal epithelial cells, and *ipaH* encodes invasion plasmid antigen H, which is involved in cell invasion. In enteroaggregative *Escherichia coli* (EAEC), the pCVD plasmid is associated with aggregative adherence [16].

Based on their genetic relatedness, *E. coli* strains are classified into phylogroups: A, B1, B2, C, D, E, F, and one specific *Escherichia* cryptic clade (clade I) [17]. Phylogrouping is essential for determining host–source relationships, characterizing extraintestinal pathogenic and commensal strains, and analyzing genetic structure [18]. Different phylogroups and pathotypes of *E. coli* have been associated with different animal‐derived foods. For example, virulence genes resembling those of human ExPEC are frequently detected in strains of *E. coli* associated with poultry meat [19].

Understanding the phylogroups and pathotypes of *E. coli* that are frequently linked to animal‐derived foods is crucial. Identifying the specific sources of ARB spreading to humans is crucial for combating the infections they cause and for developing effective treatment and prevention strategies, which remain inadequate. Only a few studies have compared different farm environments as potential sources of drug‐resistant bacteria. Furthermore, comparative studies on seasonal differences in antimicrobial resistance of *E. coli* in farm animals in Bangladesh are lacking. The present study was thus conducted to determine the seasonal variation in antimicrobial resistance in *E. coli* and to compare the prevalence of drug‐resistant *E. coli* in different farm settings. Additionally, we determined the prevalence and distribution of phylogenetic groups and pathotypes of *E. coli* in poultry, dairy, and fish farms. The aim was also to investigate the antibiotic susceptibility pattern, ESBL production, biofilm‐forming capability, antibiotic resistance genes (ARGs), and virulence and adhesin genes in different phylogroups and pathotypes.

## 2. Materials and Methods

### 2.1. Isolation and Identification of *E. coli*

Samples were collected from dairy, poultry, and fish farms in Cumilla, Bangladesh (N23°26.922 ^′^/E91°04.717 ^′^) between December, 2022, and June, 2023. Sampling was conducted during three distinct seasons: summer, rainy, and late autumn. In each season, we collected 13 different types of samples, with two samples per type. Given three seasons, the total number of samples collected was 78 (13 sample types × 2 samples per type × 3 seasons = 78 samples) (Table 1). During the collection of samples, sterile falcon tubes, gloves, cotton, swab sticks, zip bags and autoclaved PBS were used. The chicken cloacal swab was collected with sterile cotton swab and then placed into a sterile falcon tube containing sterile PBS. Soil samples were collected using a sterile spatula and then transferred into a sterile ziplock bag. The poultry drop was collected with a sterile forceps and then transferred into a sterile ziplock bag. Milk was directly collected in a sterile falcon tube from the dairy farm where they used a sterile container for milk collection. Water from the fish farm pond was collected directly using a sterile bottle. Soil or sediment was collected using sterile spatula and kept in a sterile ziplock bag.

**Table 1:** Distribution of *E. coli* isolates among different sources.

Source	Source type	Number of identified *E.coli* isolates (*n* = 63)	
Poultry farm	Poultry swab	15	33
	Soil	5	
	Feed	0	
	Poultry dropping	13	

Dairy farm	Cow dung	17	28
	Soil	10	
	Feed	1	
	Milk	0	

Fish farm	Fish	0	2
	Soil	0	
	Sediment	0	
	Pond water	2	
	Feed	0	

The collected samples were kept in the insulated icebox during transportation and at 4^o^C until processing. Solid samples (e.g., soil, feed, poultry droppings, and cow dung) were homogenized using a stomacher. PBS was used as buffer for the processing of solid samples in the stomacher. The fish sample was processed by mechanical grinding after separation of the gill, gut, and skin scale using a sterile knife. In the case of liquid samples, such as water and poultry swabs collected in PBS, were analyzed without further processing.

*E. coli* was isolated using selective media, namely eosin methylene blue (EMB) agar (Oxoid Limited, England) and MacConkey agar (Oxoid Limited, England). Initially, 0.1 ml of the processed sample was spread on EMB agar plates. After overnight incubation at 37°C, greenish colonies on EMB agar were selected for further streaking. These colonies were then streaked onto both EMB agar and MacConkey agar plates and incubated again at 37°C. The greenish colonies from EMB agar and pink colonies from MacConkey agar were further streaked onto nutrient agar plates and incubated overnight at 37°C. The colonies from nutrient agar were then subjected to both biochemical and molecular methods for further identification.

Nine biochemical tests were performed: Methyl Red (MR)‐Voges–Proskauer (VP) test, motility test, urease test, indole (MIU) test, citrate utilization test, Kligler′s Iron Agar (KIA) test, gelatin hydrolysis test, nitrate reduction test, oxidase test, catalase test, and starch hydrolysis test. At the molecular level, the isolates were identified by PCR targeting the *E. coli uidA* gene using specific primers: uidA (F): 5 ^′^‐TATGGAATTTCGCCGATTTT‐3 ^′^, *uid*A (R): TGTTTGCCTCCCTGCTGCGG as previously described [20].

### 2.2. Antibiotic Susceptibility Testing

Antibiotic susceptibility testing was performed using the Kirby–Bauer disk diffusion method. At first, bacterial isolates were cultured in Mueller–Hinton Broth (MHB) (Oxoid, United Kingdom) for 1–1.5 h to a turbidity equivalent to 0.5 McFarland standard. The bacterial lawn was prepared on Mueller Hilton agar (MHA) plates and the antibiotic disks were placed. The plates were incubated for 16–18 h at 37°C. The zones of inhibition were measured and the susceptibility was scored as recommended by the Clinical and Laboratory Standards Institute (CLSI) guidelines [21]. Commercially available antibiotic disks (Oxoid, United Kingdom) used in this study included ampicillin (10 *μ*g), ciprofloxacin (5 *μ*g), trimethoprim–sulfamethoxazole (25 *μ*g), piperacillin/tazobactam (100/10 *μ*g), gentamicin (10 *μ*g), chloramphenicol (30 *μ*g), amikacin (30 *μ*g), imipenem (10 *μ*g), meropenem (10 *μ*g), doripenem (10 *μ*g), cefuroxime (30 *μ*g), cefoxitin (30 *μ*g), cefotaxime (30 *μ*g), ceftazidime (30 *μ*g), cefepime (30 *μ*g), aztreonam (30 *μ*g), tigecycline (15 *μ*g), doxycycline (30 *μ*g), and tetracycline (30 *μ*g). The standard strain used in this study was *E. coli* ATCC25922.

The isolates showing resistance to one or more agents in at least three antimicrobial classes were characterized as MDR isolates [22]. For each isolate, the multiple antibiotic resistance index (MARI), which provides a quantitative measure of the extent of antibiotic resistance, was also estimated. It ranges from 0 (completely susceptible to all tested antibiotics) to 1 (resistant to all tested antibiotics). Higher MAR index values indicate a higher level of multiple antibiotic resistance in the isolate.

MARI=number of antibiotics to which the isolate is resistant/Total number of antibiotics tested.

### 2.3. Extended Spectrum Beta Lactamase (ESBL) Phenotype Confirmation

The production of ESBL in the isolates was assessed through both screening and confirmatory tests. Screening tests are initial assays used to identify potential ESBL producers, whereas confirmatory tests are performed to validate the presence of ESBL production. All isolates were screened for ESBL production by disc diffusion test using indicator cephalosporins, namely ceftazidime (30 *μ*g) and cefotaxime (30 *μ*g). ESBL‐producing isolates can hydrolyze cephalosporins such as ceftazidime and cefotaxime. Therefore, isolates showing resistance to at least one of these cephalosporins were considered potential ESBL producers. To confirm ESBL production, the modified double disc synergy test (MDDST) was carried out. The MDDST is a confirmatory test used to detect ESBL production in bacterial isolates [23]. By placing cephalosporin discs around an Augmentin disc and observing for synergistic increase in zone size, the presence of ESBLs can be confirmed. The antibiotic discs used in the MDDST test include ceftazidime (CAZ 30 *μ*g), cefotaxime (CTX 30 *μ*g), ceftriaxone (CRO 30 *μ*g), cefepime (FEP 30 *μ*g), and amoxicillin/clavulanic acid (AMC/CLA) (20/10 *μ*g) discs.

One colony of pure culture isolates was suspended in MHB and incubated at 37°C for 3–4 h to yield an inoculum corresponding to 0.5 McFarland standard. For each isolate, a bacterial lawn was prepared on the surface of the Mueller–Hinton agar. A disc of augmentin (20 *μ*g amoxicillin + 10 *μ*g CLA) was placed in the middle on the surface of the MHA plate, and then discs of CRO (30 *μ*g), CAZ (30 *μ*g), CTX (30 *μ*g) and FEP (30 *μ*g) were placed around it at a distance of 15 to 20 mm from the augmentin disc (center to center). The plates were incubated overnight at 37°C. The isolates were considered ESBL positive when there was any deformation or increase in the zone size towards the disc of amoxicillin–clavulanate.

### 2.4. Quantitative Biofilm Formation Assay

The ability of *E. coli* isolates to form biofilms was assessed by microtiter dish biofilm formation assay using 96‐well polystyrene plates [24]. Samples were initially cultured in Luria broth (LB) medium at 37°C for 24 h with subsequent 1:100 dilution and inoculation into microplate wells (triplicates per isolate). After overnight incubation, unattached cells and residual media were removed, and wells were gently washed with sterile water, air‐dried, and stained with 0.1% crystal violet. Following incubation and rinsing, 33% acetic acid was added to solubilize the dye‐cell complex, and absorbance at 560 nm was measured using a plate reader with a 33% acetic acid in water blank.

The optical density cut‐off value (ODc) was set as three standard deviations (SD) above the mean of the optical density (OD) of the negative control as shown in the following formula: ODc = average OD of negative control + (3 × SD of negative control). The results were summarized into four categories based on their optical densities: (1) strong biofilm producer (4 × ODc < OD); (2) medium biofilm producer (2 × ODc < OD ≤ 4 × ODc); (3) weak biofilm producer (ODc < OD ≤ 2 × ODc); and (4) nonbiofilm producer (OD ≤ ODc) [25].

### 2.5. DNA Extraction

The entire genomic DNA from *E. coli* isolates was obtained from freshly cultured bacteria using the QIAamp DNA Mini Kit (QIAGEN, Hilden, Germany) following the manufacturer′s guidelines. Subsequently, the DNA concentration was determined with the Colibri Microvolume Spectrometer (Titertek–Berthold, Berthold Detection Systems GmbH, Bleichstrasse, Pforzheim, Germany), whereas the integrity of the DNA was assessed through electrophoresis on 0.8% (w/v) agarose gels. These DNA samples were preserved at −20°C until they were used for PCR assays.

### 2.6. Detection of Adhesin Genes

Polymerase chain reaction assays for detection of *crl*, *fimH*, *fimA*, and *papC* genes were performed by a set of primers as shown in Table S1. The reaction mixture contained 6.25 *μ*l of Taq DNA Polymerase Master Mix (OneTaq Quick‐Load DNA polymerase), 0.5 *μ*l (10 PM/*μ*l) of each primer, 3.25 *μ*l of distilled water, and 2 *μ*l (100 ng) of DNA template in a final volume of 12.5 *μ*l. PCR was performed under the following conditions: 95°C for 4 min, 95°C for 60 s, primer‐specific annealing temperature for 45 s, and 72°C for 60 s for 35 cycles, with a final extension at 72°C for 7 min. PCR products were visualized using Gel Doc (BioRad, United States) following electrophoresis through 1.5% agarose gel stained with ethidium bromide.

### 2.7. Virulence Detection and Pathotyping of *E. coli* Isolates

Molecular characterization of *E. coli* pathotypes was performed based on the detection of the virulence genes of *E. coli* by PCR following established procedures [15, 26]. Genes used to screen for identification of different pathotypes include *eltB* and *estA* for ETEC, *eaeA* for atypical EPEC, *eaeA* and *bfpA* for typical EPEC, *eaeA*, *vt1*, and *vt2* for EHEC, *ial* and *ipaH* for EIEC, and *pCVD* for EAEC. A total of nine pairs of primers were used to detect the virulence genes, as listed in Table S2. The reaction mixtures were prepared as discussed in the previous section. PCR reactions were performed under the following conditions: 96°C for 4 min, 94°C for 20 s, annealing temperature for 20 s, and 72°C for 10 s for 40 cycles, with a final extension at 72°C for 7 min. PCR products were visualized, as mentioned before.

### 2.8. *E. coli* Phylogrouping

The phylogroups of the experimented isolates were determined by using a combination of four phylogroup‐specific gene primers designated as *arpA* (encode ankyrin repeat protein), *chuA* (encode an outer membrane gene required for haem transport), *yjaA* (encode a hypothetical protein), and *tspE4C2* (a DNA sequence situated within a gene encoding a putative lipase esterase [17]. In addition, two additional primers were used to detect Phylogroup C and E. The primers targeted by these markers are shown in Table S3.

### 2.9. Detection of ARGs

Based on phenotypic resistance to antibiotics, the isolates were further tested for the detection of ARGs, namely, *blaTEM*, *blaCTX-M-15*, *blaSHV*, *blaOXA-1*, *blaOXA-48*, *blaCMY-2*, *sul 1*, *sul 2*, *qnrB*, *qnrS*, *dfrA1*, *dfrA17*, *tetA*, and *aadA* by PCR. The primers used to detect the ARGs are listed in Table S4. The reaction mixtures were prepared as discussed in the previous section. PCR reactions were performed under the following conditions: 95°C for 3 min, 95°C for 45 s, primer‐specific annealing temperature for 45 s, and 72°C for 45 s for 35 cycles, with a final extension at 72°C for 7 min. PCR products were visualized, as mentioned before.

### 2.10. Statistical Analysis

Data analysis was performed using the R statistical programming language within the RStudio integrated development environment [27, 28]. The datasets were imported into RStudio for initial cleaning, processing, and statistical analysis. R packages including “tidyverse” and “ggplot2” were utilized for data manipulation and visualization [29, 30]. The frequency of ARGs in different biofilm groups and MARI variation in different seasons and in different biofilm groups were evaluated by one‐way ANOVA following the Tukey HSD post hoc test. A two‐tailed *t*‐test was used to compare MARI between EHEC and EPEC and between B1 and C phylogroups. Wilcoxon rank sum test was carried out to compare any two groups with nonnormal data. Differences were considered statistically significant when  ^∗^ *p* < 0.05. All measurements were performed in triplicate.

## 3. Results

### 3.1. Isolation, Identification, and Antibiotic Susceptibility Testing

From the sample listed in Table 1, a total of 63 strains were isolated and identified as *E. coli* by morphological and biochemical tests. The identified isolates were tested for the *E. coli* specific *uidA* gene. All 63 isolates tested positive for the *E. coli*‐specific *uidA* gene. Because of the limited number of *E. coli* isolates identified from aquaculture (*n* = 2), fish farms were not included in subsequent statistical analyses.

All isolates were tested against 19 antibiotic agents in the categories of aminoglycosides, quinolones, phenicols, cephalosporins, glycylcycline, monobactams, cephamycins, cefepime, carbapenems, penicillins, folate pathway inhibitors, antipseudomonal penicillin with *β*‐lactamase inhibitors, and tetracyclines by the Kirby–Bauer disc diffusion method using Mueller–Hinton agar. Among the 63 isolates, resistance to ciprofloxacin (55.54%) was the most common, followed by resistance to tetracycline (49.21%), doxycycline (49.21%), trimethoprim–sulfamethoxazole (42.86%), ampicillin (36.51%), amikacin (26.98%), chloramphenicol (14.29%), gentamicin (12.70%), doripenem (6.35%), cefotaxime (4.76%), aztreonam (4.76%), piperacillin‐tazobactam (3.18%), cefuroxime (3.18%), tigecycline (3.18%), imipenem (1.59%), meropenem (1.59%), cefepime (1.59%), and cefoxitin (1.59%) antibiotics, as shown in Figure 1. All the isolates were sensitive to ceftazidime (30 *μ*g). Antibiotic resistance patterns varied notably across sample types (Figure 2a). Poultry‐associated samples (swabs and droppings) exhibited the highest resistance frequencies, with poultry swabs showing resistance to over 80% of tested antibiotics including CIP, SXT, and TE. Notably, 100% resistance was observed to CXM among poultry swabs and pond water. In contrast, cow dung and dairy‐related samples displayed generally lower resistance rates, though dairy soil showed high resistance to AK (80%).

**Figure 1:**
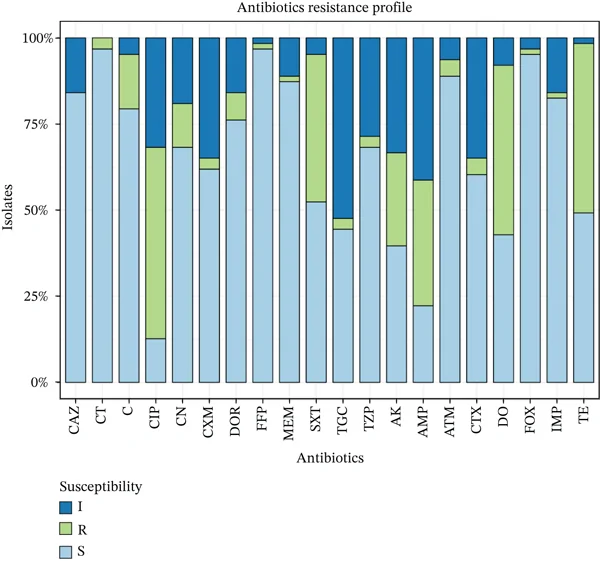
Antibiotic susceptibility profile of *E. coli* isolates. (CN, gentamicin; AK, amikacin; TZP, piperacillin‐tazobactam; IMP, imipenem; MEM, meropenem; DOR, doripenem; CXM, cefuroxime; CTX, cefotaxime; CAZ, ceftazidime; FEP, cefepime; FOX, cefoxitin; CIP, ciprofloxacin; TGC, tigecycline; SXT, trimethoprim‐sulfamethoxazole; ATM, aztreonam; AMP, ampicillin; C, chloramphenicol; TE, tetracycline; DO, doxycycline).

**Figure 2:**
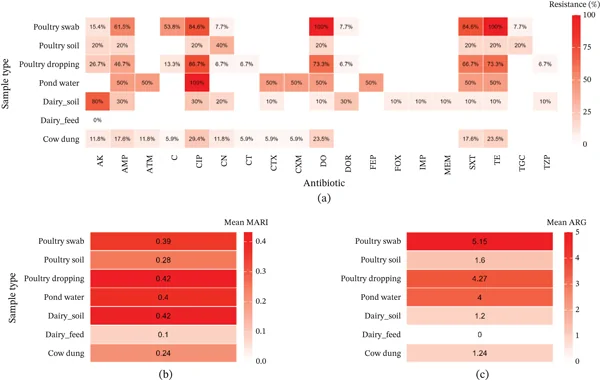
Antibiotic resistance patterns across sample types. (a) Percentage of antibiotic‐resistant isolates across different sample types. (b) Variation in mean multiple antibiotic resistance index (MARI) across sample types. (c) Variation in mean frequency of antibiotic resistance genes (ARGs) across sample types.

### 3.2. MARI

The MARI values ranged from 0.00 to 0.82 (Table 2), with 39 isolates (62%) exhibiting a MARI of ≥ 0.3. There was no significant difference in the MAR index among pathotypes, phylogroups, and isolate types (*p* − values = 0.13, 0.49, and 0.36, respectively; all >0.05), indicating an equal spread of phenotypic resistance to multiple antibiotics (Figure 3a,b,d). However, the MAR index was significantly higher in the isolates sampled during the rainy season than in the isolates from the summer season (*p* − value = 0.027 < 0.05), as shown in Figure 3c. The mean MARI values (Figure 2b) were highest in poultry droppings and dairy soil (0.42), followed by pond water (0.40) and poultry swabs (0.39). Dairy feed exhibited the lowest MARI (0.10), indicating a minimal antibiotic resistance burden.

**Table 2:** Distribution of *E. coli* in different MARI ranges.

MARI range	Number of isolates	Percentage
0.0–0.1	8	12.7%
0.1–0.2	9	14.2%
0.2–0.3	13	20%
0.3–0.4	12	19%
0.4–0.5	8	12.7%
0.5–0.6	9	14.2%
0.6–0.7	1	1.5%
0.7–0.8	3	4.8%

**Figure 3:**
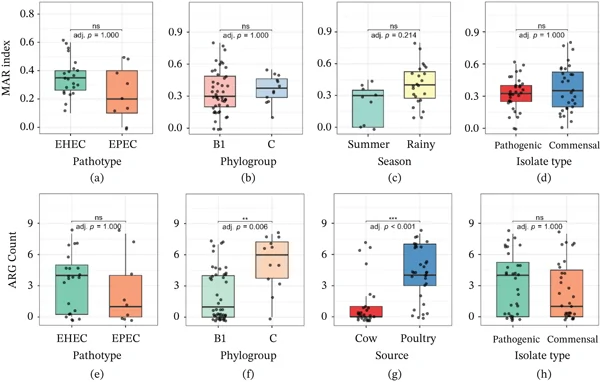
Multiple antibiotic resistance (MAR) index variation and antibiotic resistance gene (ARG) count variation across different isolate attributes: (a) MARI variation between pathotypes EHEC and EPEC; (b) MARI variation between Phylogroups B1 and C; (c) represent the seasonal variation of *E. coli* isolates; (d) MARI variation between Commensal and pathogenic isolate types; (e) ARG count variation between pathotypes EHEC and EPEC; (f) ARG count variation phylogroups B1 and C; (g) ARG count variation between isolates from cow and poultry sources; (h) ARG count variation between commensal and pathogenic isolate types. Each boxplot shows the interquartile range (IQR), median, and distribution of values with individual data points. *p*‐values from Wilcoxon rank‐sum tests are displayed above each comparison.

### 3.3. Multidrug Resistance Profile

The MAR index measures resistance to all antibiotics applied to a specific isolate, whereas the MDR percentage indicates the proportion of a bacterial group resistant to at least three out of the total antibiotics tested. The MDR percentage was determined based on antibiotic susceptibility testing. Approximately 55.56% (35 out of 63) isolates were MDR *E. coli*. The percentage of MDR isolates was also higher in the isolates sampled during the rainy season (78.26%) than those sampled during the summer (66.67%). Between sources, the prevalence of MDR isolates was more common in poultry (78.79%) than in cattle farms (25%). Among samples, the MDR percentage was highest in poultry soil (100%), followed by poultry droppings (84.62%), poultry swabs (66.67%), dairy soil (30%), and dairy dung (23.53%). Two of the isolates from aquaculture water were also MDR.

### 3.4. Phenotypic Detection of ESBL Production

MDDST was performed to confirm ESBL production in the isolates. Out of 63 *E. coli* isolates, only three were found to be positive for ESBL by the MDDST test.

### 3.5. Biofilm Formation

The biofilm‐forming ability of the isolates was evaluated by crystal violet assay. Among the 63 isolates, only 6.3% (4 isolates) were nonbiofilm producers, 81% (51 isolates) were weak biofilm formers, 7.9% (5 isolates) were moderate biofilm formers, and 4.8% (3 isolates) were strong biofilm formers (Figure 4c). Based on sample types, 100% of isolates from poultry soil, pond water, and dairy feed exhibited weak biofilm formation. Strong biofilm‐forming isolates were rare, detected only in cow dung (11.8%) and poultry swabs (7.7%) (Figure 5a). When mean OD was compared between Phylogroup B1 and C, isolates from B1 phylogroup showed significantly higher mean OD than phylogroup C (*p* value = 0.045 < 0.05), as shown in Figure 6b. However, no significant difference in mean OD was observed between EPEC and EHEC (*p* − value = 0.98 > 0.05) (Figure 6a). Similarly, no significant differences in mean OD were observed between isolates from poultry and cow sources (*p* − value = 0.26 > 0.05), or between commensal and pathogenic isolates (*p* − value = 0.23 > 0.05), as shown in Figure 6c and Figure 6d.

**Figure 4:**
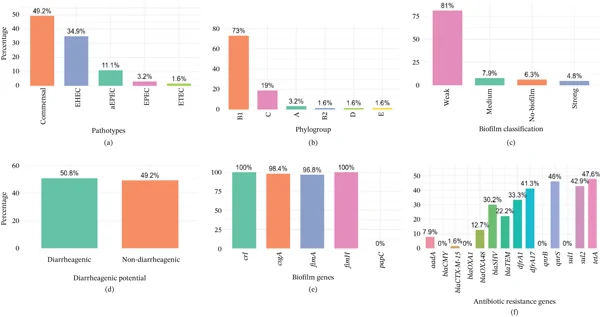
Distribution of genomic and phenotypic traits among *E. coli* isolates. (a) Distribution of pathotypes, showing predominance of commensal and EHEC types. (b) Distribution of phylogroups, with B1 as the most prevalent. (c) Biofilm classification, revealing the majority of isolates as weak biofilm formers. (d) Comparison of diarrheagenic versus nondiarrheagenic isolates. (e) Prevalence of biofilm‐associated genes (*crl*, *csgA*, *fimA*, *fimH*, and *papC*), with *crl* and *fimH* present in 100% of isolates. (f) Frequency of antibiotic resistance genes (ARGs), showing high prevalence of *tetA*, *sul2*, *qnrS*, and *blaTEM* among the isolates.

**Figure 5:**
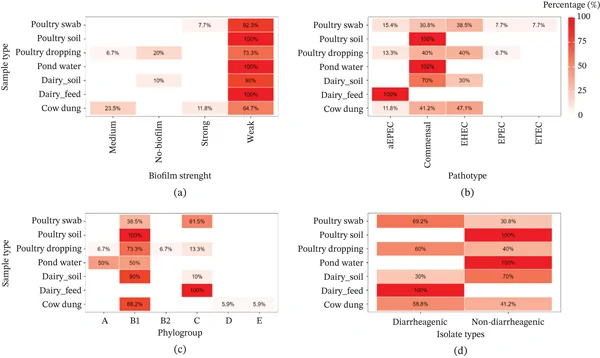
Heat map visualization of sample‐type‐specific distributions of isolate traits. (a) Distribution of biofilm strength across different sample types, showing that most isolates were weak biofilm formers, with strong biofilm formation observed only in poultry‐related samples. (b) Distribution of pathotypes by sample type, where EHEC and commensal strains predominated across cow dung, dairy, and poultry sources. (c) Distribution of phylogroups across sample types, with phylogroup B1 being the most prevalent, especially in dairy‐related and poultry environments. (d) Comparison of diarrheagenic and nondiarrheagenic isolates by sample type, indicating a higher proportion of diarrheagenic isolates in poultry and pond water sources.

**Figure 6:**
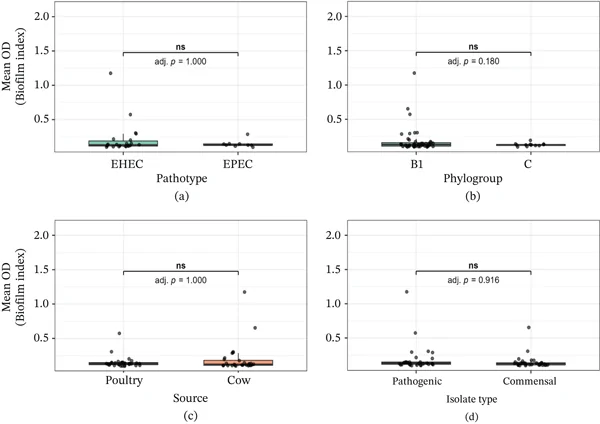
Mean OD (Biofilm Index) variation within different groups. (a) Mean OD variation between pathotypes EHEC and EPEC. (b) Mean OD variation between Phylogroups B1 and C. (c) Mean OD variation between cow and poultry. (d) Mean OD variation between pathogenic and commensal. Each boxplot shows the interquartile range (IQR), median, and distribution of values with individual data points. *p*‐values from Wilcoxon rank‐sum tests are displayed above each comparison.

### 3.6. Prevalence of Adhesin Genes

All isolates were screened for adhesin genes at the molecular level to detect adhesin genes such as *csgA*, *papC*, *fimA*, *crl*, *and fimH*. Among the five adhesin genes, *crl* and *fimH* were present in all the isolates. The prevalence of *csgA* and *fimA* was 98.41% and 96.83%, respectively. No isolate was found positive for the *papC* gene (Figure 4e).

### 3.7. Pathotyping of *E. coli*

Based on the presence and absence of pathotype‐specific genes such as *eltB*, *estA*, *eaeA*, *vt1*, *vt2*, *bfpA*, *pCVD*, *ial*, and *ipaH*, the isolates were grouped into different pathotypes. Among the isolates, Enterohemorrhagic *E. coli* (EHEC) (34.92%) was the most prevalent pathotype, confirmed by the presence of EHEC‐specific genes such as *eaeA*, *vt1*, and *vt2*, followed by atypical EPEC (11%) and typical EPEC (3.17%) (Figure 4a). Atypical EPEC was confirmed based on the presence of the *eaeA* and *bfpA* genes and the absence of the *vt1* and *vt2* genes, whereas typical EPEC was confirmed based on the sole presence of the *eaeA* gene. Only one isolate was found positive for both *eltA* and *estA* genes and was characterized as ETEC. None of the isolates tested positive for *pCVD*, *ial*, or *ipaH*, indicating the absence of EAEC and EIEC pathotypes. In our study, isolates characterized as ETEC and EPEC were only identified from poultry farm (Figure 5b).

### 3.8. Phylogrouping of *E. coli*

Based on the presence and absence of phylogroup‐associated genes such as *arpA*, *chuA*, *yjaA*, and *tspE4.C2*, the isolates were grouped into A, B1, B2, C, and D phylogroups. Among the analyzed isolates, the majority (73.02%) belonged to Phylogroup B1, followed by C (19.02%) and A (3.17%), as shown in Figure 4b. Only three isolates were characterized as Phylogroup B2, D, and E. *E. coli* strains belonging primarily to the B2 phylogroup, and to a lesser extent, the D phylogroup is recognized as the main cause of extraintestinal infections in humans. Moreover, the B2 and E phylogroups are probably associated with diarrheal disease. In our study, isolates characterized as Phylogroup D and E were isolated from a dairy farm, whereas one isolate characterized as Phylogroup B2 was isolated from a poultry farm (Figure 5c).

### 3.9. Detection of ARGs in *E. coli* Isolates

Fourteen genes associated with antibiotic resistance were screened. Among these, the distribution of *tetA* (47.62%) was the most common, followed by *qnrS* (46.03%), *sul-2* (42.86%), *dfrA17* (41.27%), *dfrA1* (33.33%), *blaSHV* (30.16%), *blaTEM* (22.22%), *blaOXA-48* (12.70%), *aadA* (7.94%), and *blaCTX-M-15* (1.59%), as shown in Figure 4f. No isolate was found positive for *blaOXA-1*, *sul-1*, *qnrB*, and *blaCMY-2* genes. As presented in Figure 3f, the prevalence of these resistance genes was significantly higher in Phylogroup C than in Phylogroup B1 (*p* − value = 0.00073 < 0.05). No significant difference in MARI was observed between the EHEC and EPEC pathotypes (*p* − value = 0.4 > 0.05) (Figure 3e), or between commensal and pathogenic isolates (*p* − value = 0.27 > 0.05) (Figure 3h). When the source was considered, isolates identified from poultry showed a significantly higher frequency of ARGs than the isolates identified from dairy (*p* − value = 0.000028 < 0.05), as shown in Figure 3g. Similarly, the mean frequency of ARG was highest in poultry swabs (5.15), followed by poultry droppings (4.27) and pond water (4.0) (Figure 2c). Dairy feed contained no detectable ARGs, and dairy soil had the lowest among ARG‐positive sources (1.2).

## 4. Discussion

The emergence and dissemination of antimicrobial resistance in *E. coli* associated with diarrheal illnesses pose a significant global health concern. Antibiotic‐resistant DEC that produce ESBL and exhibit multidrug resistance properties further exacerbate the public health threat [31]. Animal farms and their surrounding water environments are recognized as major reservoirs of various MDR DEC strains. Considering this fact, we collected samples from different farms, including dairy farms, poultry farms, and fish farms. The objective was to isolate *E. coli* and investigate their characteristics, including multidrug resistance patterns, diarrheagenic potential, virulence genes, adhesin genes, drug resistance genes, and phylogrouping. The isolation and identification of *E. coli* were achieved through conventional cultural, microscopic, biochemical, and molecular methods. For molecular identification, the *uidA* gene primer was used to detect the *β*‐d‐glucuronidase gene (*uidA*), specific to *E. coli*. Following the procedures, a total of 63 *E. coli* isolates were identified.

### 4.1. Prevalence of ARGs and Phenotypic Resistance

The antibiotic resistance profile of the isolates was determined using 19 antibiotics of 12 different classes of antibiotics. The highest resistance was observed against ciprofloxacin, with 55.56% of isolates showing resistance. Resistance to ciprofloxacin was even higher in another report from farm animals in Bangladesh, showing almost 80% resistance [32]. Our study investigated two resistance genes, *qnrS* and *qnrB*, which confer resistance to quinolones such as ciprofloxacin. Although *qnrB* was absent, 46.03% of the isolates were positive for the *qnrS* gene. Both *qnrS* and *qnrB* are examples of plasmid‐mediated resistance genes and have the potential to horizontally transfer quinolone resistance to other species [33].

The second highest resistance was found against tetracycline and doxycycline antibiotics, exerted by 49.21% of the isolates. These isolates may acquire resistance via active efflux pumps encoded by the *tetA* gene or by the production of ribosomal protection proteins (RPPs) [34–36]. When the presence of the *tetA* gene was tested by molecular detection, 47.62% of the isolates were found positive. The finding correlates with phenotypic resistance, suggesting that *tetA* gene expression may contribute to tetracycline resistance. The third highest level of resistance was observed for trimethoprim–sulfamethoxazole (42.86%). Bacteria can acquire resistance to trimethoprim and sulfamethoxazole through various mechanisms. In our study, four resistance genes, including *sul1*, *sul2*, *dfrA1*, and *dfrA17*, which usually confer resistance against trimethoprim and sulfamethoxazole, were investigated. Variants of the *dfr* gene, including *dfrA1* and *dfrA17*, produce an altered form of the dihydrofolate reductase enzyme that reduces the binding affinity for trimethoprim, whereas *sul1* and *sul2* confer resistance against sulfamethoxazole antibiotic [30, 37, 38]. Although no isolates were positive for *sul1*, the prevalence of the other three genes, *sul2* (42.86%), *dfrA1* (33.33%), and *dfrA17* (41.27%), significantly correlates with phenotypic resistance to trimethoprim and sulfamethoxazole.

Against ESBL antibiotics, the resistance is as follows: cefotaxime (4.76%), cefuroxime (3.76%), and cefepime (1.59%). There was no resistance found against ceftazidime, a 3^rd^ generation cephalosporin. We investigated the prevalence of ESBL genes, including *blaSHV*, *blaTEM*, and *blaCTX-M-15*, which are usually involved in hydrolyzing the 2^nd^, 3^rd^, and 4^th^ generation cephalosporins. Among the ESBL genes, *blaSHV* (30.16%) was the most prevalent, followed by *blaTEM* (22.22%) and *blaCTX-M-15* (1.59%). Although the prevalence of *blaSHV* and *blaTEM* is higher than the prevalence of phenotypic resistance, their presence is insufficient to confer resistance to ESBL antibiotics. However, they might enable the hydrolysis of the beta‐lactam ring in ampicillin and other beta‐lactam antibiotics, as approximately 36.51% of the tested isolates showed resistance to ampicillin. Other than these *β*‐lactamase genes, the presence of *blaOXA1* and *blaCMY* was investigated. However, no isolates were found to be positive. To reveal the phenotypic activity of ESBL, all the isolates were screened by the MDDST. Only 4.7% of isolates demonstrated the capability to produce the ESBL enzyme, which was confirmed by observing a clear enhancement towards the augmenting disc (amoxicillin + clavulanic acid). The positive isolates were from dairy and aquaculture.

Among the aminoglycosides, the isolates showed higher resistance to amikacin (26.98%) and relatively lower resistance to gentamicin (12.70%). Production of aminoglycoside‐modifying enzymes (AMEs) or modification of the ribosome by acquired 16S rRNA methyltransferases (RMTases) are the two important mechanisms by which these isolates might become resistant to aminoglycosides [39]. In this study, we investigated the presence of the *aadA* gene, which encodes aminoglycoside adenylyl transferase, an aminoglycoside modifying enzyme causing structural alteration of the aminoglycoside antibiotic [40, 41]. Our results revealed that only 7.94% of the isolates have the *aadA* gene, indicating the presence of other mechanisms of aminoglycoside resistance.

Only 14.29% of isolates showed resistance against chloramphenicol antibiotic. The resistant isolates of *E. coli* may acquire resistance mechanisms through enzymatic inactivation, primarily mediated by acetylation through acetyltransferases or chloramphenicol phosphotransferase enzymes [42, 43]. A low prevalence of resistance was observed against three carbapenem antibiotics: imipenem (1.59%), meropenem (1.59%), and doripenem (6.35%). This suggests that the prevalence of carbapenem resistance mechanisms in these isolates is insignificant, which can be viewed as a positive outcome. However, there has been a study with no report of carbapenem‐resistant *E. coli* from poultry environments in Bangladesh [44]. This implies a growing resistance to carbapenems in *E. coli* present in the farm environments.

### 4.2. Multidrug Resistance Profile

MDR *E. coli* in farm animals has become a significant public health concern [45]. In our study, 55.56% of the isolates were characterized as MDR *E. coli*. Notably, MDR isolates were more prevalent in poultry (78.79%) compared with dairy (25%). The high presence of MDR *E. coli* in poultry has also been reported in other studies conducted in Bangladesh [46, 47]. A similar result was observed in the prevalence of ARGs; isolates identified from poultry showed a significantly higher frequency of ARGs than those identified from dairy (*p* − value = 0.019 < 0.05). The high prevalence of MDR *E. coli* in poultry farms can likely be attributed to several factors. These include the practice of raising a large number of chickens in a confined space, minimal spacing between the chickens, and the mixing of residual feed and water with chicken manure. These conditions create an environment that promotes the spread and proliferation of MDR *E. coli* strains.

Seasonal changes may also have a significant impact on the prevalence of MDR *E. coli*. In this study, we found the highest percentage of MDR isolates, precisely 78.26% (18 out of 23), in the rainy season, followed by 66.67% (6 out of 9) during summer and 35.48% (11 out of 31) during late autumn. Similarly, the MAR index was significantly higher in the isolates sampled during the rainy season than those from the summer (*p* − value = 0.027 < 0.05). The occurrence of optimal growth conditions, increased transmission of ARGs, humidity, rainfall, temperature, and sanitation practices are believed to be key factors contributing to the elevated prevalence of MDR *E. coli* during both the summer and rainy seasons [45].

### 4.3. Biofilm Formation

Biofilm formation in *E. coli* has been associated with increasing antibiotic resistance in several studies [48–50]. A crystal violet assay was performed to reveal the isolates′ phenotypic biofilm‐forming capability. Approximately 93.6% (strong 4.8%, moderate 7.9%, and weak 81%) of the isolates demonstrated biofilm‐forming ability. Only three isolates were strong biofilm formers, among which two were classified as MDR isolates. Infection caused by these strong biofilm‐forming isolates will have little sensitivity toward many broad‐spectrum antibiotics. Biofilm formation was also investigated among different phylogroups and pathotypes of *E. coli*. Although there is no definitive evidence indicating that a specific pathotype or phylogroup consistently forms stronger biofilms, we observed variations in biofilm formation capabilities. Specifically, our findings revealed that biofilm formation was significantly higher in Phylogroup B1 compared with Phylogroup C (*p* value = 0.045 < 0.05). All the strong biofilm formers were from Phylogroup B1. However, no significant difference in biofilm formation was observed among the different pathotypes (*p* value = 0.22 > 0.05).

### 4.4. Prevalence of DEC

DEC are frequently isolated and identified in different farm settings [45]. In our study, 50.8% of the isolates were identified as DEC, which was mostly from poultry and dairy (Figure 4d). Based on sources, 54.55% of the poultry isolates and 50% of the dairy isolates were identified as DEC, whereas no DEC was found in the aquaculture sample (Figure 5d). This finding suggests that the environment of poultry farms has a higher likelihood of harboring DEC because of the high population density of chickens and the potential contamination of water and feed. A similar trend was observed in the case of dairy farms, indicating a strong association between DEC and ruminant animals. Seasonal variation may also impact the prevalence of DEC. We found DEC more common in the summer (66.67%) and rainy seasons (65.22%) and less common in late autumn (33.33%).

The threat posed by DEC becomes more severe when they acquire MDR characteristics, as they can pose a potential risk of treatment failures in patients with diarrhea. In our study, 59.38% of the identified DEC exhibited multidrug resistance. The high prevalence of MDR *E. coli* suggests an urgent need for surveillance measures to control the dissemination of MDR strains from farm animals.

### 4.5. Distribution of Pathotypes

Most of the *E. coli* pathotypes found in the environment are derived from dairy and poultry waste. These pathogens have the ability to survive and spread in various farm environments, including compost, grazing soil, and water bodies, from where they can further disseminate through rainfall and various other means. This poses a risk of transmission to other warm‐blooded hosts, including humans [51].

In this study, pathotype screening was conducted using molecular techniques to investigate their distribution. The molecular analysis revealed that the most prevalent pathotype was EHEC (34.92%), confirmed by the presence of specific genes such as *eaeA*, *vt1*, and *vt2*. The *eaeA* gene encodes intimin, a 94‐kDa outer membrane protein involved in the intimate attachment of EHEC and EPEC to mammalian host cells [52, 53]. Among the isolates, 33.33% were positive for *vt1*, followed by 9.52% positive for *vt2*. These genes encode verotoxins, also known as Shiga toxins, which play a significant role in the pathogenesis of EHEC [14]. Nine isolates were positive for the *eaeA* gene but tested negative for both the *vt1* and *vt2* genes. These isolates may belong to either typical EPEC or atypical EPEC. Among nine isolates, only two were positive for *bfpA* genes, indicating their classification as typical EPEC. Moreover, seven isolates tested positive only for the *eaeA* gene but showed negative results for *vt1*, *vt2*, and *bfpA* genes, indicating their classification as atypical EPEC. These atypical EPEC isolates also lacked the EPEC adherence factor plasmid (pEAF) in their structure.

Only one isolate from poultry was positive for the heat‐labile toxin gene (*elt*), confirmed by observing a 322 bp amplicon size. The presence of the *elt* gene indicated that the isolate was ETEC. This suggests a low presence of ETEC (1.59%) compared with EHEC and EPEC in this study. In contrast to our findings, ETEC was relatively more common than EHEC in a study carried out on livestock in Bangladesh [54]. The absence of the *ipaH* and *ial* genes suggests the absence of EIEC, whereas the absence of *pCVD* suggests the nonappearance of EAEC.

### 4.6. Distribution of Phylogroups

The phylogroups of the experimented isolates were determined by using a combination of four phylogroup‐specific gene primers designated as *arpA* (encodes ankyrin repeat protein), *chuA* (encodes an outer membrane gene required for haem transport), *yjaA* (encodes a hypothetical protein), and *tspE4C2* (a DNA sequence situated within a gene encoding a putative lipase esterase) [55]. In addition, two additional primers were used to detect Phylogroup C and E.

Among the isolates analyzed in this study, the majority, comprising 73.02% of the isolates, belonged to the B1 phylogroup, following Phylogroup C (19%). Phylogroups A, B2, D, and E had lower representation, with 3.17% isolates in Phylogroup A and only one isolate each (1.59%) in Phylogroup B2, D, and E. Similar to our findings, previous studies in Bangladesh have also found Phylogroup B1 to be predominant [56]. Similar findings have been reported in farm animals in other countries as well [57, 58].

### 4.7. Diversity of Adhesin Genes

Biofilm formation in *E coli* is an intricate process that entails the expression of several genes. In this study, the prevalence of five different adhesin genes was investigated. The two most prevalent adhesin genes were *fimH* and *crl*, which were present in all the isolates. The *fimH* gene encodes a bacterial adhesin protein located at the tip of *E. coli* fimbriae, contributing to bacterial adherence to host cells and enhancing pathogenicity [59]. The *crl* gene, a curli regulatory gene, controls the expression of *curli* and plays a role in specific adherence and pathogenesis [60].

The third most prevalent gene was *csgA*, a curlin subunit gene found in 98.41% of the isolates. Curli proteins are essential for biofilm formation, cell invasion, and the overall phenotype of *E. coli* strains [61]. The high prevalence of *csgA* correlates with the biofilm‐forming ability observed in approximately 94% of the isolates. Furthermore, the *fimA* gene, another Type 1 fimbria gene, was detected in 96.83% of the isolates. However, the *papC* gene associated with P fimbriae in uropathogenic *E. coli* (UPEC) was not detected in any of the isolates, indicating the absence of the UPEC strain.

## 5. Conclusion

Our study predominantly found B1 phylogroup *E. coli* strains in poultry and dairy environments. We observed the prevalence of MDR DEC, particularly the EHEC pathotype. An interesting relation between the rainy season and the prevalence of MDR *E. coli* was observed. Our results revealed that poultry is the major source of these threatening isolates. The strains identified in our study have biofilm‐forming ability alongside drug resistance. We suggest that ceftazidime can be used to treat or eliminate *E. coli* isolates in farm environments. We also found considerable efficacy of carbapenems against these strains, which can be used as an alternative to ceftazidime. However, further research should be conducted to investigate the mechanisms of resistance, the effectiveness of alternative treatments, and the potential for implementing comprehensive control strategies to mitigate the spread of these MDR *E. coli* strains in farm environments.

## Author Contributions

Elias Mia designed and conducted the experiment, and contributed to manuscript writing. Tasnimul Arabi Anik wrote the manuscript and analyzed the data. Afroja Yasmin assisted with manuscript writing. Humaira Akhter reviewed the manuscript and provided constructive suggestions for preparation. Anowara Begum supervised the research experiment, served as the principal investigator, and reviewed the manuscript.

## Funding

This study was supported by the University Grant Commission, University of Dhaka.

## Ethics Statement

Sample collection from animal sources (e.g., poultry swabs and cow dung) and subsequent processing were conducted in accordance with institutional and national ethical guidelines. The sampling procedures were reviewed and approved by the Ethical Review Committee of the Faculty of Biological Sciences, University of Dhaka (Approval No. 290/Biol. Scs.). The authors confirm that all methods were carried out in compliance with relevant ethical regulations and guidelines.

## Conflicts of Interest

The authors declare no conflicts of interest.

## Supporting information


**Supporting Information** Additional supporting information can be found online in the Supporting Information section. Table S1: The primers used in this study for detection of adhesin genes. Table S2: The primers used in this study for detection of pathotypes. Table S3:The primers used in this study for detection of phylogroups. Table S4: The primers used in this study for detection of antibiotic resistance genes. Table S5: Biochemical profile of the isolates.

## Data Availability

The important data is already available in the manuscript. Any further datasets generated during and/or analyzed during this study if needed are available from the corresponding author on reasonable request.
