# Supplementary material for: Farm environments: Reservoir for Multidrug‐Resistant and Virulent Escherichia coli in Bangladesh
Source: Int J Microbiol. 2026 Oct 29;2026:3646267. doi: 10.1155/ijm/3646267 (PMC13720354; doi:10.1155/ijm/3646267)
Supplement: Supplementary file 1 [file IJM-2026-3646267-s001.zip › 3646267.f1/Supplementary_Table_1.docx]

**Supplementary Table 1** The primers used in this study for detection of adhesin genes

| Adhesin genes | Primer name | Primer sequence (5′-3′) | Amplicon size (bp) | Annealing T(°C) | References |
| --- | --- | --- | --- | --- | --- |
| *csgA* | csgA F  csgA R | ACTCTGACTTGACTATTACC  AGATGCAGTCTGGTCAAC | 200 | 48 | 63 |
| *crl* | crl F  crl R | TTTCGATTGTCTGGCTGTAT  CTTCAGATTCAGCGTCGTC | 250 | 59 |  |
| *fimH* | fimH F  fimH R | TGCAGAACGGATAAGCCGTGG  GCAGTCACCTGCCCTCCGGTA | 508 | 55 | 64 |
| *fimA* | fimA F  fimA R | CGACGCATCTTCCTCATTCTTCT  ATTGGTTCCGTTATTCAGGGTTGTT | 721 | 60 | 65 |
| *papC* | papC F  papC R | GACGGCTGTACTGCAGGGTGTGGCG  ATATCCTTTCTGCAGGGATGCAATA | 328 | 60 | 66 |
