# Supplementary material for: Farm environments: Reservoir for Multidrug‐Resistant and Virulent Escherichia coli in Bangladesh
Source: Int J Microbiol. 2026 Oct 29;2026:3646267. doi: 10.1155/ijm/3646267 (PMC13720354; doi:10.1155/ijm/3646267)
Supplement: Supplementary file 1 [file IJM-2026-3646267-s001.zip › 3646267.f1/Supplementary_Table_2.docx]

**Supplementary Table 2** The primers used in this study for detection of pathotypes

| Target gene | Primer name | Primer sequence (5′-3′) | Amplicon size (bp) | Annealing T(°C) | References |
| --- | --- | --- | --- | --- | --- |
| *eltB* | LT-F  LT-R | TCTCTATGTGCATACGGAGC  CCATACTGATTGCCGCAAT | 322 | 54 | 67 |
| *estA* | ST-F  ST-R | GCTAAACCAGTAGAGGTCTTCAAAA  CCCGGTACAGAGCAGGATTACAACA | 147 | 55 |  |
| *vt1* | vt1-F  vt1-R | GAAGAGTCCGTGGGATTACG  AGCGATGCAGCTATTAATAA | 130 | 55 |  |
| *vt2* | vt2-F  vt2-R | ACCGTTTTTCAGATTTTGACACATA  TACACAGGAGCAGTTTCAGACAGT | 298 | 55 |  |
| *eaeA* | eaeA-F  eaeA-R | CACACGAATAAACTGACTAAAATG  AAAAACGCTGACCCGCACCTAAAT | 376 | 57 |  |
| *ial* | Shig-F  Shig-R | CTGGTAGGTATGGTGAGG  CCAGGCCAACAATTATTTCC | 320 | 55 |  |
| *bfpA* | bfpA-F  bfpA-R | TTCTTGGTGCTTGCGTGTCTTTT  TTTTGTTTGTTGTATCTTTGTAA | 367 | 55 |  |
| *pCVD* | EA-F  EA-R | CTGGCGAAAGACTGTATCAT  CAATGTATAGAAATCCGCTGTT | 630 | 55 |  |
| *ipaH* | ipaH-F  ipaH-R | GCTGGAAAAACTCAGTGCCT  CCAGTCCGTAAATTCATTCT | 424 | 55 | 68 |
