# Supplementary material for: Farm environments: Reservoir for Multidrug‐Resistant and Virulent Escherichia coli in Bangladesh
Source: Int J Microbiol. 2026 Oct 29;2026:3646267. doi: 10.1155/ijm/3646267 (PMC13720354; doi:10.1155/ijm/3646267)
Supplement: Supplementary file 1 [file IJM-2026-3646267-s001.zip › 3646267.f1/Supplementary_Table_3.docx]

**Supplementary Table 3** The primers used in this study for detection of phylogroups

| Target genes | Primer name | Primer sequence (5′-3′) | Amplicon size (bp) | Annealing T(°C) | References |
| --- | --- | --- | --- | --- | --- |
| *chuA* | chuA.1b  chuA.2 | ATGGTACCGGACGAACCAAC  TGCCGCCAGTACCAAAGACA | 288 | 59 | 78 |
| *yjaA* | yjaA.1b  yjaA.2b | CAAACGTGAAGTGTCAGGAG  AATGCGTTCCTCAACCTGTG | 211 | 59 |  |
| *TspE4.C2* | TspE4C2.1b  TspE4C2.2b | CACTATTCGTAAGGTCATCC  AGTTTATCGCTGCGGGTCGC | 152 | 59 |  |
| *arpA* | AceK.f  ArpA1.r | AACGCTATTCGCCAGCTTGC  TCTCCCCATACCGTACGCTA | 400 | 59 |  |
| *trpA* | trpAgpC.1  trpAgpC.2 | AGTTTTATGCCCAGTGCGAG  TCTGCGCCGGTCACGCCC | 219 | 59 |  |
| *arpA* | ArpAgpE.f  ArpAgpE.r | GATTCCATCTTGTCAAAATATGCC  GAAAAGAAAAAGAATTCCCAAGAG | 301 | 57 |  |
