# Supplementary material for: Farm environments: Reservoir for Multidrug‐Resistant and Virulent Escherichia coli in Bangladesh
Source: Int J Microbiol. 2026 Oct 29;2026:3646267. doi: 10.1155/ijm/3646267 (PMC13720354; doi:10.1155/ijm/3646267)
Supplement: Supplementary file 1 [file IJM-2026-3646267-s001.zip › 3646267.f1/Supplementary_Table_4.docx]

**Supplementary Table 4** The primers used in this study for detection of antibiotic resistance genes.

| Target genes | Primer name | Primer sequence (5′-3′) | Amplicon size (bp) | Annealing  T(°C) | References |
| --- | --- | --- | --- | --- | --- |
| *bla_TEM_* | TEM F  TEM R | TCGGGGAAATGTGCGCG  TGCTTAATCAGTGAGGCACC | 972 | 60 | 69 |
| *bla_CTX-M-15_* | CTX-M15 F  CTX-M15 R | CACACGTGGAATTTAGGGACT  GCCGTCTAAGGCGATAAACA | 996 | 56 | 70 |
| *bla_SHV_* | SHV F  SHV R | CCAGCAGGATCTGGTGGACTAC  CCGGGAAGCGCCTCAT | 231 | 60 | 71 |
| *bla_OXA-1_* | OXA-1 F  OXA-1 R | ACACAATACATATCAACTTCGC  AGTGTGTTTAGAATGGTGATC | 814 | 54 | 70 |
| *bla_OXA-48_* | OXA-48 F  OXA-48 R | GCGTGGTTAAGGATGAACAC  CGCTCCGATACGTGTAACTT | 307 | 55 | 72 |
| *bla_CMY-2_* | CMY-2 F  CMY-2 R | TGGCCGTTGCCGTTATCTAC  CCCGTTTTATGCACCCATGA | 870 | 55 | 73 |
| *sul 1* | Sul1 F  Sul1 R | GTGACGGTGTTCGGCATTCT  TTTACAGGAAGGCCAACGGT | 668 | 58 | 74 |
| *sul 2* | Sul-2 F  Sul-2 R | GGCAGATGTGATCGACCTCG  ATGCCGGGATCAAGGACAAG | 405 | 58 | 74 |
| *qnrB* | QnrB F  QnrB R | GGMATHGAAATTCGCCACTG  TTTGCYGYYCGCCAGTCGAA | 264 | 54 | 75 |
| *qnrS* | QnrS F  QnrS R | GCAAGTTCATTGAACAGGGT  TCTAAACCGTCGAGTTCGGCG | 428 | 54 | 74 |
| *dfrA1* | dfrA1 F  dfrA1 R | ACGGATCCTGGCTGTTGGTTGGACGC  CGGAATTCACCTTCCGGCTCGATGTC | 254 | 55 | 76 |
| *dfrA17* | dfrA 17 F  dfrA 17 R | GTCGCCCTAAAACAAAGTTA  CGCCCATAGAGTCAAATGT | 195 | 53 | 76 |
| *tetA* | TetA F  TetA R | GTAATTCTGAGCACTGTCGC  CTGCCTGGACAACATTGCTT | 956 | 58 | 77 |
| *aadA* | aadA-F  aadA-R | CAGCGCAATGACATTCTTGG  ATCCTTCGGCGCGATTTTG | 282 | 60 | 77 |
