# Supplementary material for: Farm environments: Reservoir for Multidrug‐Resistant and Virulent Escherichia coli in Bangladesh
Source: Int J Microbiol. 2026 Oct 29;2026:3646267. doi: 10.1155/ijm/3646267 (PMC13720354; doi:10.1155/ijm/3646267)
Supplement: Supplementary file 1 [file IJM-2026-3646267-s001.zip › 3646267.f1/Supplementary_Table_5.docx]

**Table-5: Biochemical profile of the isolates**

| Biochemical tests | Result | Comment | Presumptive bacteria |
| --- | --- | --- | --- |
| Methyl Red (MR) test | Positive (+) | Bacteria fermented glucose and lowered the pH of the media by producing acid. | ***Escherichia coli***  ***Escherichia coli*** |
| Voges-Proskauer (VP) test | Negative (-) | The isolates were not able to convert glucose into neutral end products. |  |
| Motility, Indole, Urease (MIU) test | Motility: Positive (+) | Isolates degraded tryptophan to indole, showed diffused growth and did not possess urease enzyme. |  |
|  | Indole: Positive (+) |  |  |
|  | Urease: Negative (-) |  |  |
| Citrate test | Negative (-) | Bacteria did not use citrate as a sole carbon source. |  |
| Kligler's Iron agar (KIA) test | Glucose: Positive (+) | Isolates fermented both glucose and lactose, produced CO_2_ gas and did not produce H_2_S gas. |  |
|  | Lactose: Positive (+) |  |  |
|  | H_2_S production: Negative (-) |  |  |
|  | Gas production: Positive (+) |  |  |
| Gelatin hydrolysis test | Negative (-) | Isolates did not have gelatinase enzyme. |  |
| Nitrate reduction test | Positive (+) | Nitrate was reduced to nitrite by bacteria with the help of nitrate reductase enzyme. |  |
| Oxidase test | Negative (-) | Isolates did not contain cytochrome oxidase enzyme. |  |
| Catalase test | Positive (+) | Isolates detoxified H_2_O_2_ into H_2_O and O_2_ by dint of catalase enzyme. |  |
| Starch hydrolysis test | Negative (-) | Isolates did not use starch molecule |  |
